# Diagnostic delay of pulmonary nontuberculous mycobacterial infection in China

**DOI:** 10.1186/2049-6958-9-48

**Published:** 2014-09-11

**Authors:** Hui Jing, Wanming Tan, Yunfeng Deng, Dachuan Gao, Liang Li, Zhiming Lu, Edward A Graviss, Xin Ma

**Affiliations:** Katharine Hsu International Research Center of Human Infectious Diseases, Shandong Provincial Chest Hospital, Shandong University, Jinan, Shandong 25013 China; Clinical Center on TB, Chinese Center for Disease Control and Prevention, Beijing, 101149 China; Department of Laboratory Medicine, Shandong Provincial Hospital, Jinan, Shandong 250021 China; The Center for Molecular and Translational Human Infectious Diseases Research, Houston Methodist Hospital Research Institute, Houston, Texas 77030 USA

**Keywords:** China, Infection, Nontuberculous mycobacterium

## Abstract

**Background:**

Nontuberculous mycobacteria (NTM) infection is an emerging, but neglected public health concern in China.

**Findings:**

To investigate diagnostic delay of NTM diseases in China, we analyzed 91 patients with pulmonary NTM infection in ShandongProvince. The median diagnostic delay time of the analyzed patients was 84 days, which was significantly associated with rural inhabitance (135 days vs. 73 days of urban inhabitance, p < 0.01) and lower level of first visiting hospitals/clinics (70 and 82 days of tertiary and secondary hospitals/clinics respectively vs. 120 days of primary hospitals/clinics, p < 0.05). *M. farcinogenes* was isolated from a 79-year-old male patient, which is the first report of pulmonary infection in humans.

**Conclusions:**

Our results indicate a significant diagnostic delay of NTM diseases in China, especially for rural patients with limited access to higher-level healthcare services.

## Introduction

Nontuberculous mycobacteria (NTM) diseases have been neglected in most of developing world [1]. With the global pandemic of HIV/AIDS and aging population, NTM have been increasingly associated with pulmonary diseases in humans [2]. In China, *Mycobacterium tuberculosis* (MTB) still causes the majority of pulmonary mycobacteria diseases. Nevertheless, in the last decade, the NTM isolation rate has shown an increasing trend in China [3, 4]. Pulmonary NTM diseases may share clinical signs with TB, causing a clinical dilemma in early diagnosis and treatment. Delay or misdiagnosis of NTM diseases may worsen the disease and increase the mortality and economic burden of patients. In this study, we retrospectively investigated the diagnostic history and clinical characteristics of patients with pulmonary NTM diseases to better understand the factors related to the diagnostic delay in China.

### Study population and methods

This study was conducted in Shandong Province, which is the second largest province in China (population size, 96 million and 59% rural) and has approximately 40,000 new TB cases annually. Shandong Provincial Chest Hospital (SPCH) is the only provincial-level hospital specialized in TB (a referral hospital) and other lung infections, and networks 139 county/city-level TB clinics. Between January 1, 2007 and December 31, 2012, 91 patients were diagnosed with pulmonary NTM disease based on the American Thoracic Society/Infectious Disease Society of America (ATS) diagnostic criteria [5]. Multiple (≥ = 2) sputum specimens from each patient were collected for Acid Fast Bacillus (AFB) test and mycobacterial culture at SPCH. Identification of mycobacteria species was first carried out at the ISO15189-certified SPCH TB reference laboratory by conventional biochemical tests, P-nitrobenzoic acid (PNB) and 2-Thiophene carboxylic acid hydrazide (TCH) testing following a standard protocol [6], and further identified by 16S rRNA gene sequence analysis (MicroSeq ID Microbial Indentification Software V2.0, PE Applied Biosystems) at the species level as previously described [7]. To minimize possible species identification error, DNA extraction and 16S rRNA gene sequencing of each NTM isolate was independently conducted twice. Statistical analysis was carried out by using SPSS (IBM, USA). The study protocol was approved by the SPCH Institutional Review Board.

## Results

Over the six study years, a total of 91 patients (mean age ± SD, 45.7 ± 16.2 years; age range, 15 – 79 years; male, 73.6%) were identified with pulmonary NTM infection from 4541 patients with positive mycobacteria cultures at SPCH, indicating an overall NTM isolation rate of 2.0% among all mycobacterial isolates, that did not show a significantly increasing or decreasing trend within the study period. The patients were predominantly male (73.6%) and adult (98.9% older than 18 years; and 37.4% from age group of 46–64 years) (Table 1). The diagnostic delay of NTM patients was defined as the period from the date of the patient's first visit at the healthcare service to the date of diagnosis. Median diagnostic delay time for the 91 NTM patients was 84 days (range, 28 – 1,144 days), and it was not significantly associated with sex and age of NTM patients (Table 1). Patients living in rural areas showed a significantly longer diagnostic delay time than those in the urban areas (135 vs. 73 days, p < 0.01). Patients with a first visit at tertiary and secondary hospitals/clinics had significantly shorter delay time than those at primary hospitals/clinics (70 and 82 days respectively vs. 120 days, p < 0.05) (Table 1).

**Table 1:** **Characteristics and diagnostic delay of 91 NTM patients**

Patient characteristics	N. (%) (N. = 91)	Median diagnostic delay (Days)	p
Sex			
Male	67 (73.6)	86	
Female	24 (26.4)	77	0.86
Age (years)			
1-17	1 (1.1)	101	
18-25	19 (20.9)	66	
26-45	23 (25.3)	81	
46-65	34 (37.4)	91	
>65	14 (15.3)	99	0.71
Inhabited areas			
Urban	61 (67.0)	73	
Rural	30 (33.0)	135	<0.01
First visiting hospitals/clinics			
Tertiary	45 (49.5)	70	
Secondary	20 (22.0)	82	
Primary	26 (28.5)	120	<0.05

All 91 NTM patients were HIV seronegative. Other comorbidities included diabetes (9.9%), cardiovascular diseases (13.2%), chronic obstructive pulmonary disease (COPD, 12.1%), and bronchiectasis (20.9%) (Table 2). *Mycobacterium (M.) intracellulare* infection accounted for 46.1% of these NTM diseases, following by *M. chelonae-abscessus* complex (28.6%), *M. kansasii* (12.1%), *M. gordonae* (5.5%), *M. fortuitum* (5.5%), *M. asiaticum* (1.1%), and *M. farcinogenes* (1.1%). In this study, a 79-year-old male patient was diagnosed with severe COPD, bronchiectasis, and pulmonary infection Positive results were shown in six consecutive sputum AFB smear tests. Mycobacterial culture and identification revealed isolation of *M. farcinogenes.* It is the second report of human infection and the first report of pulmonary *M. farcinogenes* disease in humans [8].

**Table 2:** **Clinical characteristics of patients infected with different NTM species**

	***M. intracellulare***	***M. chelonae/abscessus complex***	***M. kansasii***	***M. gordonae***	***M. fortuitum***	***M. asiaticum***	***M. farcinogenes***	Total
N (%)	42 (46.1)	26 (28.6)	11 (12.1)	5 (5.5)	5 (5.5)	1 (1.1)	1 (1.1)	91 (100.0)
**Symptoms**								
Chest pain/distress	10 (23.8)	14 (53.8)	1 (9.1)	0 (0.0)	2 (40.0)	1 (100.0)	0 (0.0)	28 (30.8)
Cough	36 (85.7)	18 (69.2)	9 (81.8)	4 (80.0)	4 (80.0)	0 (0.0)	1 (100.0)	72 (79.1)
Hemoptysis	8 (19.0)	6 (23.1)	6 (54.5)	2 (40.0)	1 (20.0)	0 (0.0)	0 (0.0)	23 (25.3)
Fever/Sweats	21 (50.0)	16 (61.5)	1 (9.1)	1 (20.0)	1 (20.0)	0 (0.0)	1 (100.0)	41 (45.1)
Comorbidities								
**Comorbid conditions**								
Diabetes	4 (9.5)	2 (7.7)	1 (9.1)	1 (20.0)	1 (20.0)	0 (0.0)	0 (0.0)	9 (9.9)
Cardiac disease	6 (14.3)	0 (0.0)	2 (18.2)	2 (40.0)	2 (40.0)	0 (0.0)	0 (0.0)	12 (13.2)
COPD	8 (19.0)	2 (7.7)	0 (0.0)	0 (0.0)	0 (0.0)	0 (0.0)	1 (100.0)	11 (12.1)
Bronchiectasis	11 (26.2)	5 (19.2)	0 (0.0)	0 (0.0)	1 (20.0)	1 (100.0)	1 (100.0)	19 (20.9)
**Chest Radiography**								
Location in lung								
Bilateral	36 (85.7)	19 (73.1)	9 (81.8)	3 (60.0)	3 (60.0)	1 (100.0)	1 (100.0)	72 (79.1)
Simply left lung	2 (4.8)	2 (7.7)	1 (9.1)	1 (20.0)	1 (20.0)	0 (0.0)	0 (0.0)	7 (7.7)
Simply right lung	4 (9.5)	5 (19.2)	1 (9.1)	1 (20.0)	1 (20.0)	0 (0.0)	0 (0.0)	12 (13.2)
Diffuse lung (up and lower lobes)	20 (47.6)	13 (50.0)	4 (36.4)	1 (20.0)	3 (60.0)	0 (0.0)	0 (0.0)	41 (45.1)
Infiltrates	36 (85.7)	21 (80.8)	8 (72.7)	4 (80.0)	5 (100)	0 (0.0)	1 (100.0)	75 (82.4)
Cavitation	28 (66.7)	8 (30.8)	10 (90.9)	2 (40.0)	3 (60.0)	1 (100.0)	1 (100.0)	53 (58.2)
Nodules	30 (71.4)	18 (69.2)	11 (100)	3 (60.0)	4 (80.0)	1 (100.0)	0 (0.0)	67 (73.6)
Miliary pattern	0 (0.0)	0 (0.0)	0 (0.0)	0 (0.0)	0 (0.0)	0 (0.0)	0 (0.0)	0 (0.0)
Consolidation	11(26.2)	4 (15.4)	1 (9.1)	0 (0.0)	1 (20.0)	0 (0.0)	1 (100.0)	18 (19.8)
Pleural effusion	4 (9.5)	5 (19.2)	0 (0.0)	0 (0.0)	2 (40.0)	0 (0.0)	1 (100.0)	12 (13.2)
Pleural thickness	11 (26.2)	8 (30.8)	3 (27.3)	0 (0.0)	1 (20.0)	1 (100.0)	0 (0.0)	24 (26.4)
Adenopathy	20 (47.6)	6 (23.1)	2 (18.2)	0 (0.0)	1 (20.0)	0 (0.0)	1 (100.0)	30 (33.0)

## Discussion

Our data suggest that the diagnosis of pulmonary NTM disease is significantly delayed in China, which is attributed to rural inhabitance, and limited access to higher-level healthcare and laboratory services (tertiary and secondary). In China, approximately 71.3% of patients with active TB live in the resource-poor rural areas, where the mycobacteria culture and species identification tests are not routine testing for each TB or NTM suspect yet [8]. In our previous studies in Shandong, without species identification testing, NTM disease was often misdiagnosed as multi-drug resistant TB (MDR-TB) and accounted for 30.7% of reported MDR-TB (due to natural resistance of NTM to anti-TB drugs) [4]. Approximately 4% of retreated TB cases were caused by NTM infections [4]. The 2010 Chinese National TB Surveillance Program has reported a NTM isolation rate of 22.9% among 363 culture positive TB suspects [9]. This data strongly indicates that NTM infection has become a significant public health concern in China, which should not be neglected in disease control practice. The clinicians and public health professionals in primary care settings need to improve their awareness of this emerging disease. Mycobacteria culture and species identification tests need to be routine testing for each TB or NTM suspect.

This study is a hospital-based surveillance study with a limited sample size, which aims to investigate the diagnostic delay caused by the healthcare system. There are definitely some other factors, such as patients’ comorbid diseases and environmental risks [10], also contributing to the delay of care, which need to be investigated in future studies.
